# Modelling regional futures at decadal scale: application to the Kimberley region

**DOI:** 10.1038/s41598-019-56646-x

**Published:** 2020-01-21

**Authors:** Fabio Boschetti, Hector Lozano-Montes, Brad Stelfox

**Affiliations:** 1 Commonwealth Scientific and Industrial Organisation, Canberra, Australia; 2 ALCES Group, Calgary, Alberta Canada

**Keywords:** Environmental impact, Socioeconomic scenarios

## Abstract

We address the question of how to provide meaningful scientific information to support environmental decision making at the regional scale and at the temporal scale of several decades in a network of marine parks in the Kimberley region of Western Australia. Where environmental sustainability is affected by slow-dynamics climate change processes and one-off investments in large infrastructure which can affect a region for decades to come, both strategic and reactive planning is necessary and prediction becomes as urgent as standard adaptive management. At the interface between future studies, socio-economic modelling and environmental modelling, we define 18 scenarios of economic development and climate change impacts and five management strategies. We explore these potential futures using coupled models of terrestrial and marine ecosystem dynamics. We obtain a projection of the Kimberley marine system to the year 2050, conditional on the chosen scenarios and management strategies. Our results suggest that climate change, not economic development, is the largest factor affecting the future of marine ecosystems in the Kimberley region, with site-attached species such as reef fish at greatest risk. These same species also benefit most from more stringent management strategies, especially expansion of sanctuary zones and Marine Protected Areas.

## Introduction

The Kimberley region of Western Australia comprises ~420,000 km^2^ of land area and ~320,000 km^2^ of marine waters (including waters out of to the edge of EEZ) with a population of ~40,000. It is renowned for its remoteness, physical beauty, pristine ecosystems, diverse biota, complex coastline, and rich Aboriginal history. Large portions of the marine region are recognised as part of a conservation reserve network, including national parks and indigenous protected areas. It also possesses considerable natural resources in terms of minerals and offshore/onshore hydrocarbons, food production (agriculture, fishing and aquaculture) and a growing tourism industry. Both State and regional governments are committed to balance growth in population, economic activity and land, coastal and marine uses to ensure employment and improved standard of living for its current and future population with environmental and social objectives, including preservation of the natural heritage and the cultural values of its Aboriginal population.

In 2012 the Australian government established the North-west Network Marine Parks to protect one of the world’s most ecologically diverse marine areas^1^. These national parks (Kimberley, 80 Mile Beach, Roebuck Bay, Ashmore Reef, Argo-Rowley Terrace) are located in Commonwealth waters (3–200 NM). The Kimberley also includes the Great Kimberley Marine Park (a network of six marine parks) declared by the state government of Western Australia (2018) located up to 3 nautical miles from the coast (80 Mile Beach, Lalang-garram/Camden Sound, Yawuru Nagulagun/Roebuck Bay, North Lalang-garram, Lalang-garram/Horizontal Falls, and North Kimberley) (see Supplementary Fig. S1). The main zoning and rules of activities within these marine parks are described in^2^. To support coastal planning and reserve management, and to address the bio-physical, ecological and social processes affecting the Kimberley marine environment, the Western Australia Marine Science Institute (WAMSI) tasked our team with integrating information relevant to long term (up to the year 2050) management and decision-making, with a specific focus on the management of the network of marine parks.

Three features of this task represent a challenge for the use of the standard conservation method of adaptive management^3^. First, economic development in the sparsely populated, remote and resource-rich Kimberley region will depend largely on one-off decisions regarding investment in large infrastructure such as roads, ports, mining sites and off-shore rigs. Once built, this infrastructure remains in place for decades, cannot be moved, and is amenable to only minor modifications. It can impose path-dependence on subsequent regional development and is not suitable to adaptive management because beyond the control of local management authorities^3^. Second, one of the key management instruments currently available in the marine environment is the establishment of Marine Protected Areas (MPAs). This is subject to complex political processes and usually depends on one-off favourable circumstances largely disconnected from the normal functioning of adaptive management cycles. As a result, while adaptive management techniques are suitable for the management of existing MPAs, they are not generally appropriate for the designation of new MPAs. Thirdly, the effects of climate change–one of the key stressors on the system in the future–are likely to unfold on a time scale too slow to provide appropriate feedbacks for adaptive management. It is thus clear that the management of the Kimberley marine environments and the network of marine parks will need to address issues which are under control of local management authorities as well as issues and processes which are beyond such control. Since the latter are not suitable for adaptive management^3^, our approach complements the traditional approach to adaptive management by borrowing from the Future Studies and Foresight literature. We suggest that in this type of conservation problems, Future Studies and adaptive management have complementary roles. The former allows us to imagine and assess long-term consequence of decisions which are hard to reverse or processes which are beyond our control, while adaptive management allows us to adjust, improve upon and mitigate such changes.

First, we involved the project’s stakeholders in defining the stressors and sources of uncertainty which were perceived to have the largest impact on the future of the region. There was general consensus that the two key drivers were climate change and population growth/economic development, so our analyses explored scenarios representing various intensities of these two factors. This resulted in 18 scenarios as described in Section 4.2 and include low, medium and high climate change; low, medium and high development (see Table 1); plus two scenarios, dry and wet precipitation conditions, needed to capture uncertainty in predicted precipitation regimes. Next, we asked stakeholders to define a set of available management strategies (management means) able to achieve the stated aspirations of ensuring environmental sustainability together with economic growth and resilience to climate change (management end). Finally, we used computer modelling to explore the dynamics of biophysical and socioeconomic processes under this set of scenarios and management strategies for the future. The outputs of our models represent the projection of the Kimberley marine system to the year 2050 conditional on the chosen scenarios and management strategies.

**Table 1:** Brief description of the Development scenarios.

Development scenarios	Low	Medium	High
Average population growth/year	1.5%	2%	2.5%
Cropland Area (1,000 ha) (Ord River Basin by mid-century)	~40	~60	~100
Cattle - heads by mid-century (average growth/year)	600 K (0%)	1.1 M (1.25%)	1.24 M (1.5%)
Roads by mid-century	As current	• Paving Cape Leveque Hwy • Upgrade Gibbs River Rd	• Upgrade Gibbs River Rd • an increase in the number of roads to the coast, or the upgrading of existing tracks • upgrade or the Kalumburu Rd
Tourism (Tourism Activity Days -TADs by mid-century)	7.7 M (1.5**%** growth)	9.8 M (2**%** growth)	12.5 M (2.5**%** growth)
Oil (m^3^/yr) & LNG (peak Mtpa) by mid-century	As current	~400 k Blina & Ungani Fields ~7.5 Browse Basin & Concerto	~600 k Blina & Ungani Fields ~10 Browse Basin & Concerto

Unless otherwise specified, growth is expressed as annual means.

While the focus of the Kimberley Marine Research Program is the establishment and management of marine parks, it has been clear from the inception of our project that the impact of terrestrial processes on marine environments also had to be accounted for in order to provide useful management advice at a regional scale. To address this, our analyses included both a marine ecosystem model (Ecopath with Ecosim, or EwE^4^) and a terrestrial model (ALCES^5^) (see Methods for a description of these models.) To our knowledge, this is one of the first applications of the outputs of a spatially explicit terrestrial ecosystem model to drive a spatially explicit marine model.

Our approach is similar to adaptive management in the involvement of stakeholders in the definition of ends and means of management processes. However, it differs in two important ways. First, it shifts emphasis from the use of the adaptive cycle of implementation, evaluation, and modification, to the use of Future Studies approaches based on scenario analysis (see above). Second, it places stronger requirements on the computer models because of both the spatial and temporal scales involved in the model projections. Well established, state-of-the-art model approaches and accurate parametrisation become even more essential than in standard modelling tasks. Here, we use well validated models to establish climate change as the most significant factor affecting the future course of marine ecosystems in the Kimberley region. We also identify groups of marine organisms at greatest risk from environmental change, and show that well designed and planned marine protected areas can mitigate at least some of these risks.

## Results

### ALCES results

The output of the ALCES model provided time series (2015–2050) of key drivers such as human population growth, changes in areal extent of wetlands and estuaries, in sediment yield transported by rivers, and in water quality and quantity due to human activities. Projections of future population using low (1.5%/yr), medium (2.0%/yr), and high (2.5%/yr) growth rates suggest that the Kimberley region’s population will grow from its current ~40,000 individuals to ~60,000 ~120,000 by 2050, with concomitant increases in demand for housing, food, water, and electricity. Planned and proposed expansions of crop agriculture, grazing, and mineral and hydrocarbon extraction will likely contribute significantly to future environmental impacts.

This information was integrated into the EwE marine ecosystem model as forcing functions affecting marine primary production rates, consumption rates and natural and fishing mortalities. The ALCES model predicted future trajectories of terrestrial habitat quality as a function of climate, grazing, crop agriculture, and other parameters. These projections, in turn, affected freshwater runoff and nutrient loading into the estuarine and marine environments and were used as ecological drivers affecting key parameters in EwE. For example, changes in terrestrial wetland extent based on human activity and climate change were used in EwE as ecological forcing with proportional effects on natural mortality, vulnerability to predation, and relative feeding rates of species associated directly with wetlands, such as migratory shorebirds, seabirds, juvenile fishes, prawns, oysters and estuarine fishes. Similarly, predicted growth in tourist pressure led to proportional changes in recreational fishing mortality, resulting in increments of mortality rates of 1.5% year^−1^ (total 68% for low growth), 2% year^−1^ (total 99% for medium growth) and 2.5% year^−1^ (total 137% for high growth) over the projection period (2015–2050). Supplementary Table S1 shows in detail the variation of the EwE input biomasses based on changes of habitat, distribution and productivity predicted by the ALCES model, as well as by expected climate change impacts on Australian fish stock due to warming, acidification and sea level rise^6^.

**Figure 1:**
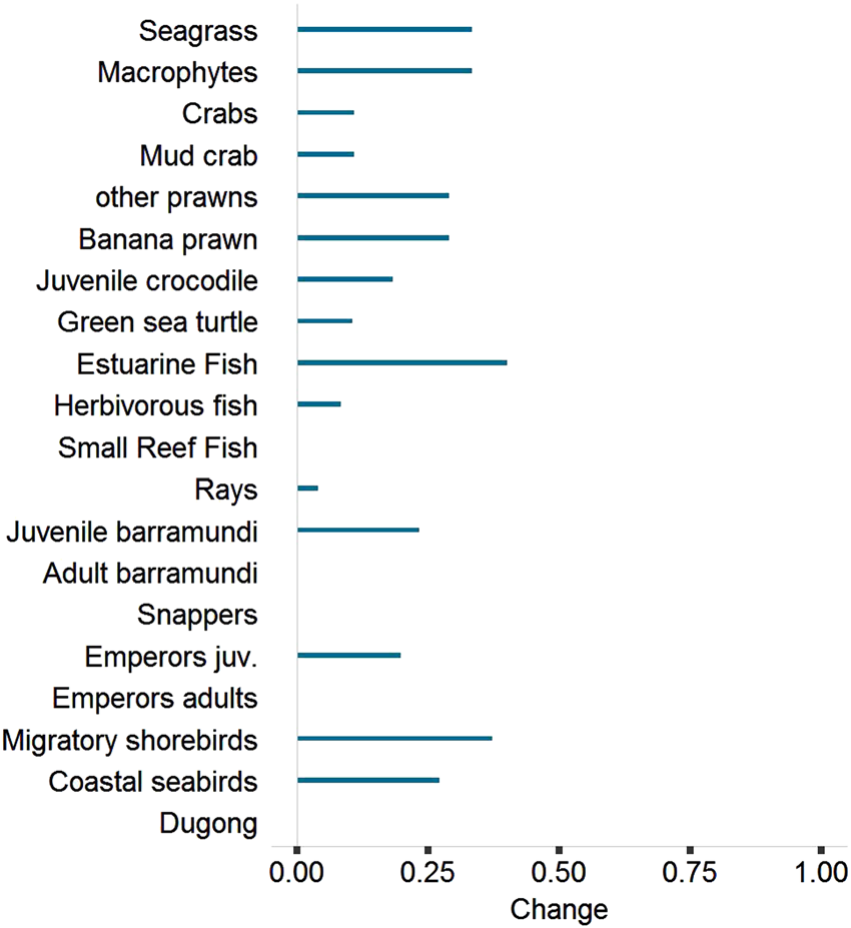
Ratio of change of biomass at the end of the simulation (2050) of the high climate, high development, wet scenario accounting for ALCES input vs the same scenario *not* accounting for ALCES input (zero means no change).

In at least some scenarios, incorporating terrestrial inputs from the ALCES model significantly changed the outcome of the EwE marine simulations. To show this, we compared the output of EwE with vs without ALCES input for the high climate impact, high development, wet scenario. For this scenario, ALCES predicted a 17.9% gain in wetlands and estuaries at 2050, due to higher precipitations in the wet scenario. For comparison, we ran the same scenario in EwE with all terrestrial forcing removed. Incorporating ALCES input changed final biomass by ~30–40% for several functional groups (seagrass, macrophytes, banana prawns, other prawns, estuarine fishes, juvenile barramundi and birds), with smaller changes for additional groups (crabs, green sea turtles, juvenile crocodiles and herbivorous fish) (Fig. 1).

### Dynamics of the marine ecosystem

**Figure 2:**
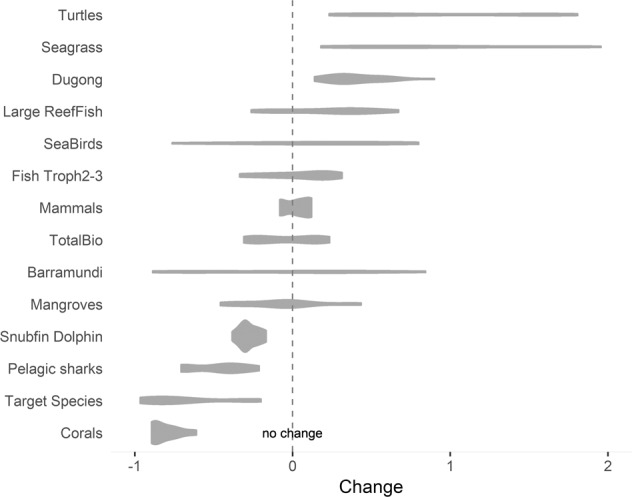
Violin plot of the state of all indicators (y axis) at the end of simulation (2050), expressed as ratio of change over the value at the beginning of the simulation (2015) (x axis). For each indicator, the violin plot shows the probability density of the data at different values, aggregated across all scenarios.

Figure 2 shows the change in biomass of functional groups and other model output which we used as system indicators (see Methods, below) over the 35 years of the EwE simulations. The plot aggregates the outcomes across all 18 climate change/development scenarios under the status quo management strategy. Values < 0 (>0) imply decrease (increase) in biomass over the simulated timespan.

The indicators can be grouped into four types according to the location and width of their distributions:

1. Winners: these are the indicators for which the distribution lays completely to the *right* hand side of the dashed “no change” line in Fig. 2, implying that biomass changes are >0 for all scenarios. These include seagrass, turtles and dugongs, likely as these species feed on seagrasses.
2. Losers: these are the indicators for which the distribution lays completely to the *left* hand side of the “no change” line in Fig. 2, implying that biomass changes are <0 for all scenarios. These include target (fish) species, corals, snubfin dolphins and pelagic sharks.
3. Low scenario-sensitivity: these are the groups whose final biomass is not much affected by the different scenarios, as shown by a narrow plot. These include: mangroves, corals, snubfin dolphins and mammals. These groups may be winners, losers or show little change at all, the important consistent feature is that their biomasses converge to a particular level regardless of the scenario; and
4. High scenario-sensitivity: the groups whose final biomass is significantly affected by the different scenarios, as shown by a wide plot. These include: seagrass, turtles and seabirds.

The response of each indicator to each specific scenario is provided in Supplementary Fig. S2.

**Figure 3:**
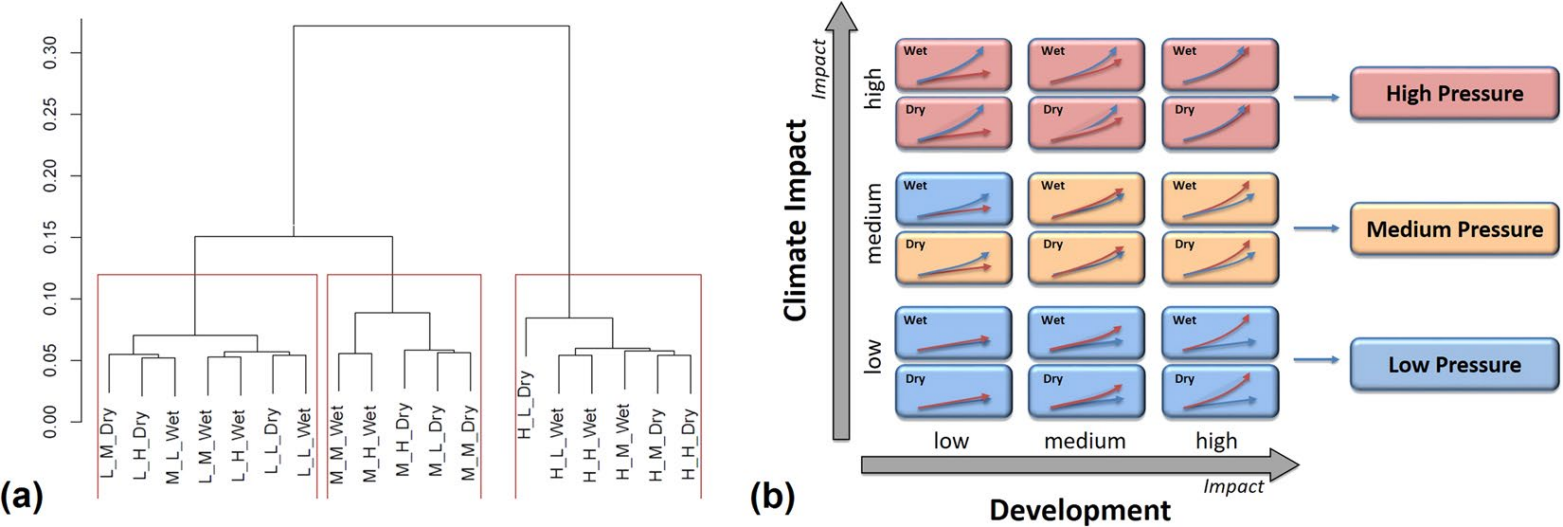
Result of the hierarchical clustering applied to the 18 scenarios (3 climate scenarios * 3 development * 2 precipitation regimes). (**a**) The cluster dendrogram suggests that the scenarios can be grouped into 3 clusters. (**b**) The 6 × 3 Future plane consisting of the Climate Change and Development axes. Each axis is subdivided into low, medium, and high impact. In addition, each cell on the plane shows ‘drier’ and a ‘wetter’ sub-scenarios. Mapping the 3 clusters over the Future’s plane clearly shows how the scenario clusters are controlled mainly by climate forcing in terms of warming.

Next, we focus on individual scenarios. To explore whether the 18 modelled scenarios for climate change and human development fall into natural groupings, we performed cluster analysis using Total Divergence^7^ (see Supplemental Material) in biomass at the end of each scenario (Fig. 3a). The 18 scenarios fall into three distinct clusters (Fig. 3b), as supported by bootstrap analysis (see Supplementary Note).

The Future plane (Fig. 3b) is controlled mainly by climate change (y axis in Fig. 3b). Within this main layering, forcing due to the precipitation regimes affects the middle cluster by assigning the Medium Climate - Low Development - Wet Precipitation scenario to the Low Climate cluster. Forcing due to socio-economic development (x axis) does not affect the clusters. As a result, our analyses of the impact of different management strategies will focus on the three climate-change clusters. In particular, we selected one scenario to represent each cluster, which we referred to as ‘High’ (High Climate, High Development, Dry Precipitation), ‘Medium’ (Medium Climate, Medium Development, Dry Precipitation) and ‘Low Pressure’ (Low Climate, Low Development, Wet Precipitation).

**Figure 4:**
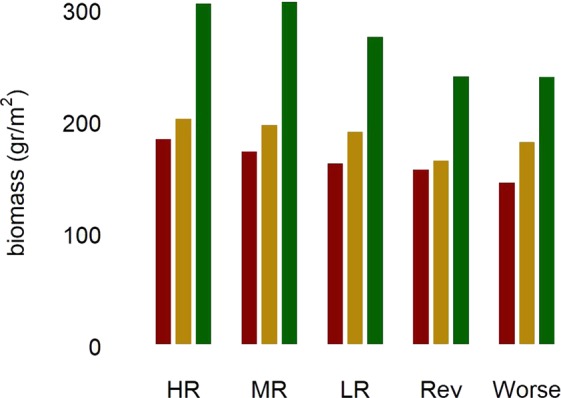
Simulated changes in the absolute total biomass at the end of simulations (2050) under different management strategies. HR = high regulation; MR = medium regulation; LR = low regulation (status quo); Rev = reversal of current conservation regulation; Worst = collapse of all regulation (see Methods for further description). Red, yellow and green bars represent ‘High’, ‘Medium’ and ‘Low’ pressure, respectively.

Next, we assessed the impact of management strategies. None of the management strategies (see Methods) significantly altered the assignment of indicators to “winner” and “loser” categories based on total marine biomass in 2050 (data not shown). More stringent conservation management (i.e. increase in the area of no take MPAs, see ‘Sanctuary Zone extension’ in Table 2) led to a small but definite increase in total biomass under all levels of climate pressure by 2050 (Fig. 4).

**Table 2:** Description of the proposed Management Strategies with regards to MPAs, Sanctuary Zone extension, fishing regulations and other human uses.

Management Tools/Regulation pressure	High	Medium	Low	Reversed	Worst Case
80 Mile Beach Marine Park, Lalang-garram/Camden Sound Marine Park, Yawuru Nagulagun/Roebuck Bay Marine Park, North Lalang-garram Marine Park, Lalang-garram/Horizontal Falls Marine Park, North Kimberley Marine Park (see Supplementary Fig. S1)	Yes	Yes	Yes	Yes	No
80 Mile Beach Commonwealth Marine Reserve, Roebuck Commonwealth Marine Reserve, Kimberley Commonwealth Marine Reserve (see Supplementary Fig. S1)	Yes	Yes	No	No	No
Sanctuary Zone extension (% of total park area)	30%	20%	10%	0	0
Fishing regulation (% virgin biomass harvested)	20%	50% (prawns) 50% (finfish)	90% (prawns) 70% (finfish)	90% (prawns) 70% (finfish)	90% (prawns) 70% (finfish)
Fish size limits	Current fish size (status quo)	Current fish size (status quo)	Status quo + ~10 cm	Status quo + ~15 cm	No limit
Bag size limits	Current bag size (status quo)	2 * Current bag size	5 * Current bag size	10 * Current bag size	10 * Current bag size
Accepted cumulative tourism-induced mortality (see caption)	0.3%	1%	5%	No limit	No limit
Accepted cumulative mortality (see caption) from other marine uses	0.3%	1%	5%	No limit	No limit

The ‘accepted cumulative tourism-induced mortality’ (second last row) includes overall mortality due to presence of tourism in remote region as a result of pollution from boats, human presence on reefs/coastline, etc. The ‘accepted cumulative mortality’ (last row) includes overall mortality due to other human uses, including Oil and Gas exploration and extraction, due to pollution, infrastructure, boat collisions, etc.

**Figure 5:**
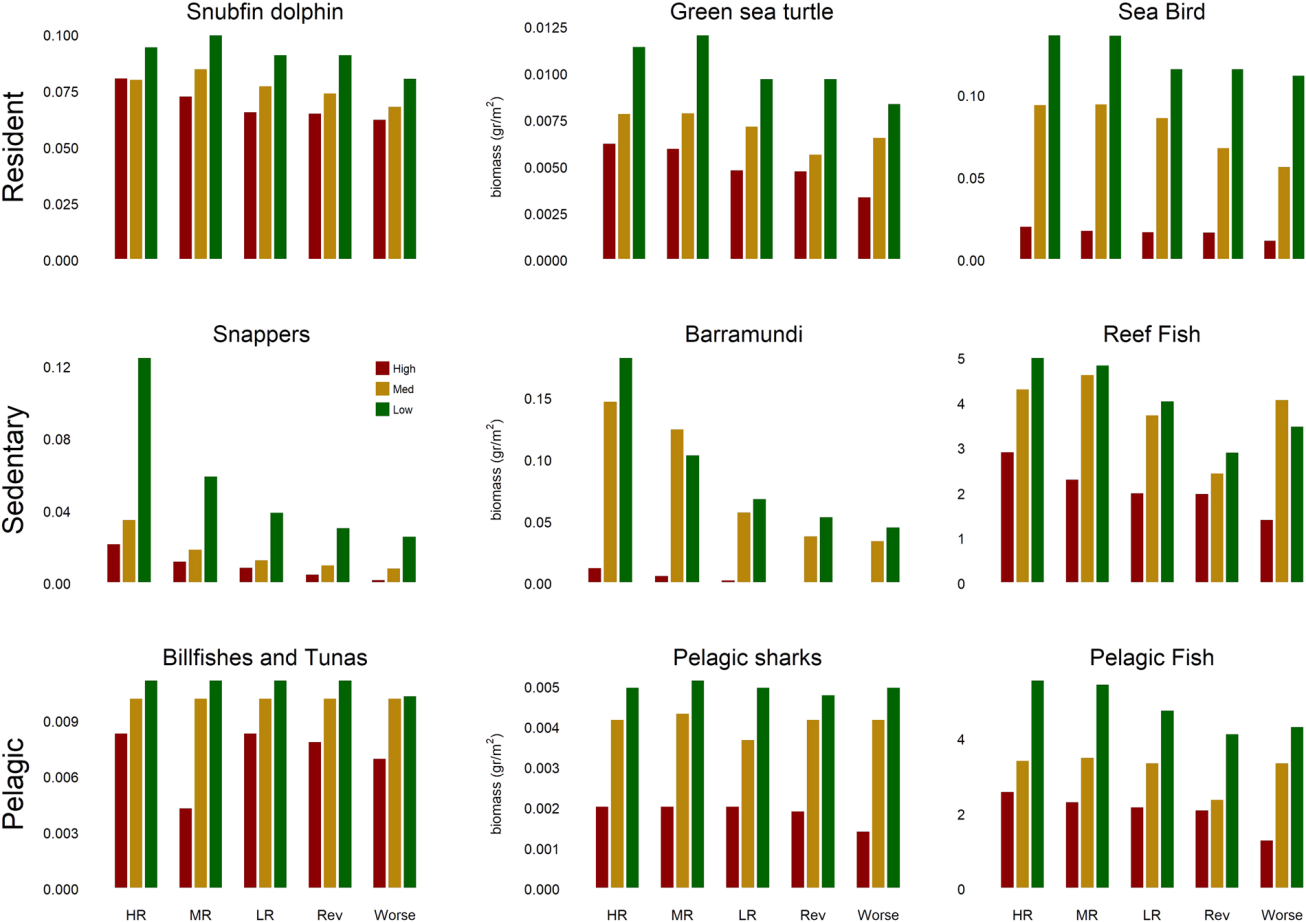
Simulated changes in the absolute biomass of selected indicators at the end of simulations (2050) under different management strategies. Red, yellow and green bars refer to ‘High’, ‘Medium’ and ‘Low’ pressure, respectively. Management strategies as in Fig. 4.

Figure 5 shows biomass changes for specific functional groups of management significance, and Supplementary Fig. S3 shows biomass changes for all indicators for each management strategy. While increased regulation appears to benefit all groups to at least a small degree, the effect of more stringent regulation is much stronger for certain groups, notably site/habitat attached species such as reef fishes. For many groups, the main factor affecting biomass in 2050 was climate change. For example, biomass was predicted to be severely reduced under the High pressure scenario, especially for snappers, barramundi, and seabirds. Nevertheless, within each scenario, management strategies may still play a crucial role in allowing the preservation of species such as snappers and barramundi above levels which would otherwise see their biomass decrease below critical levels.

More stringent management strategies include both larger and more numerous MPAs and more restrictive fishing regulations (Table 2). To show that MPAs are an important management tool, we compared model output biomass with and without the MPA network under the same fishing regulations for the high climate, high development, dry precipitation scenario (see Supplementary Fig. S4). Biomass of most species decreases in the absence of MPAs. Species showing the largest effect are barramundi, snappers, emperors, and dugongs. In contrast, their prey show an increase in biomass in the absence of MPAs.

## Discussion

Our study couples a detailed spatial model of the impacts of terrestrial land use (ALCES) to a similarly detailed spatial model of marine trophic networks (EwE). Few other studies have integrated terrestrial and marine dynamics^8–10^. Our results show that even for the sparsely populated Kimberley region, where the anthropogenic footprint is extremely light, incorporating the effects of terrestrial land use can alter model outputs of the estimated biomass of marine species by 30 to 40% for some groups. We would expect land use to have more noticeable effects on marine systems at a local scale, particularly at or near river mouths and near the centres of population or industrial activity, which are beyond the scope of this study.

**Figure 6:**
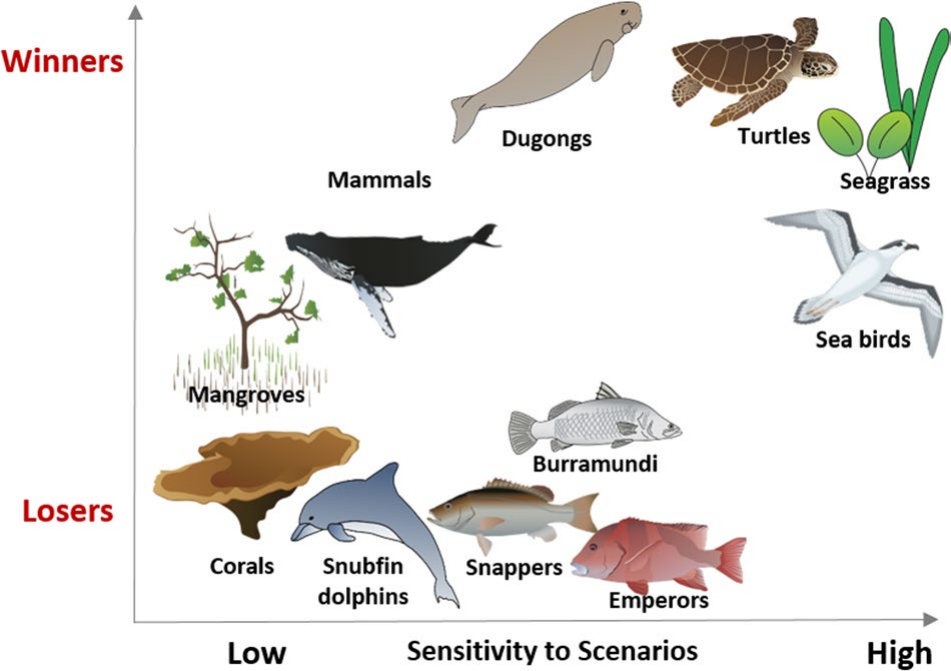
Plot of some functional groups over ‘sensitivity to scenario’ vs ‘winners-losers’ axes. The ‘sensitivity to scenario’ axis indicates how much the response of a functional group varies between different scenarios. The ‘winners-losers’ axis shows the extent to which the group biomass increases or decreases during the simulation as a function of the scenario. Symbols courtesy of the Integration and Application Network (ian.umces.edu/symbols/).

Our results suggest that different portions of the marine ecosystem may respond differently to different climate and development pressures. By the year 2050, the state of some groups (e.g., seagrass, turtles, and dugongs) varies dramatically from scenario to scenario, while other groups (e.g. corals, snubfin dolphins, mammals, mangroves) show little variation (Fig. 6, x axis). These outputs may be used to develop monitoring and management priorities in the future. For example, groups predicted to be most sensitive to different climate and development pressures should have high priority for long-term monitoring for two reasons. First, this will improve our knowledge of key aspects of these groups’ life history, such as changes in habitat ranges, recruitment, growth and survival rates. This will lead to better model parameterisation and thus an improved understanding of the factors driving these sensitivities. Second, the sensitivity of some marine groups to climate change provides a good indicator for the early detection of system responses that may help identify which, among the modelled scenarios, the system is heading towards^11,12^.

Some functional groups (corals, snubfin dolphins, pelagic sharks) are consistently losers and others (seagrass, dugongs and turtles) are consistently winners under a wide variety of scenarios. In contrast, the performance of other groups depends considerably on the precise scenario which may eventuate (Fig. 6, y axis). These results can provide managers with an indication of the expected direction, magnitude, and consistency of a group’s response to changing climate or development and thus the extent to which a management intervention targeted at a specific group is likely to succeed. Of particular note, corals show pronounced declines in biomass under all climate change scenarios, in some cases falling below 20% of 2015 biomass. Corals could struggle to survive to 2050 under a high climate change scenario unless some means can be found to increase their adaptive capacity, either through the natural survival and reproduction of more heat tolerant individuals and/or human intervention, as has been proposed for the Great Barrier Reef^13,14^.

Our comparisons of the outcomes from different climate change and development scenarios suggested that climate change was the primary factor affecting the future course of ecosystem response. The climate scenarios modelled in this work are the ones recommended by the Intergovernmental Panel on Climate Change (IPCC)^15^. These represent expected warming under different pathways of future anthropogenic CO_2_ emissions. While there is uncertainty on which pathway will materialise (as well as on the level of warming produced by a given pathway^16^), anthropogenic CO_2_ emissions change slowly and their impact on the climate has a delayed response^16^. This means that the likelihood of occurrence of each modelled scenario can be assessed in advance and will become better defined in future years. This has two implications: i) CO_2_ emission changes will likely occur smoothly and therefore, time for contingency planning may be available and ii) the impact of management actions will also be slow and subject to system inertia, which supports strategic, rather than reactive, management.

The analysis of the available management options we have explored (Figs. 4 and 5) suggests that a 20% to 30% increase in Sanctuary Zone extension over present levels would lead to an increase in total system biomass under most scenarios. More specifically, Sanctuary Zone extension can be particularly beneficial to exploited groups such as Barramundi (*Lates calcarifer*), Snappers (e.g. *Lutjanus* spp) and Emperors (e.g *Lethrinus* spp) and for relatively sedentary species such as reef fishes (e.g. *Choerodon* spp, *Scarus* spp, *Cheilinus* spp). These results suggest that sanctuary zones within marine parks may be an important tool to meet conservation objectives in the face of changing climate and human impacts.

Large knowledge gaps still exist which, if addressed, could considerably improve our models’ forecasts. In particular, little information currently exists about the biology of many species (e.g. juvenile fish, turtle hatchlings, pelagic fish, deep demersal fish and mud crab). This leads to uncertainty about how climate change will affect life history dynamics, geographic ranges, or the timing of migration or spawning of these species.

Our models also do not incorporate the effect of ocean acidification on the marine ecosystems of the Kimberley. Currently, little information is available on the impacts or time course of ocean acidification, particularly in the Kimberley region. To complicate things further, responses to ocean acidification are variable among species, including closely related species, requiring caution when trying to generalise data among species^17^.

Overall, climate change is forecast to produce negative effects on the total biomass, diversity and productivity of the Kimberley system by 2050. It also serves as an “accelerant of instability”^18^ because uncertain precipitation regimes lead to unpredictable variation in sediment runoff and areas of wetlands, estuaries, and mangroves^1^.

Our study shows the utility of scenario analysis and ecological modelling as a complement to adaptive management in guiding decisions about the designation of MPAs as a conservation response to climate change. As mentioned in the Introduction, the establishment of the network of marine parks in the Kimberly region and the research supporting its management has several purposes, which include the protection of the local ecosystems, charismatic species, ecosystem services and cultural values. As currently recognised in the literature^19,20^, this is in line with increasing expectations of the functions protected areas provide and the impacts and pressures they address. Some of these impacts may be under the direct control of management authorities as resulting from local human footprint, including fishing and tourism in our study. Other pressures are beyond the control of local management authorities. In our study, this applies to climate change, whose control lays at the global scale, and one-off infrastructure development and public and political appetite for environmental conservation, whose control lays at national and international scales. In^19^, it is acknowledged that protected areas are often designed to addresses the effects (direct drivers), not the causes (indirect drivers) of change. Within this framework, in our work direct drivers are addressed via adaptive management of the network of marine parks. By protecting key coastal habitats such as coral reefs, seagrass beds, mangroves and wetlands, MPAs help both maintaining the resilience of marine ecosystems to climate variability^20,21^ and lessening the impact of non-climate change stressors such as water pollution, overfishing and habitat destruction^22^. In our work, the model outputs show that sedentary finfish species such as Barramundi, emperors, snappers and reef fishes are likely to show the greatest benefits from expanded MPAs.

Where the causes of change in terms of indirect drivers cannot be addressed by local MPAs, our approach employs scenario development to understand the global context and uncertainty within which local management operates^3^. By involving stakeholders both in imagining future scenarios at the global and regional scale and considering options for adaptive managements at the local scale, our approach helps bringing to the fore the interlinked, multiscale social–ecological nature of the problems we address^19^.

## Methods

### The models

The ALCES model (www.alces.ca) is a landscape/land-use simulator suitable to explore the cumulative effects of land uses (residential, transportation, croplands, livestock, mining, oil and gas, forestry, tourism/recreation) and natural disturbances regimes (fire, landslides, storms, climate, climate change) upon a comprehensive set of economic, social, and environmental outcomes. For each of these processes, ALCES also tracks resource production and supply using approaches that are typical to the specific sector. The model uses a map-based representation (5 × 5 m grid) layered with geospatial data on land uses (including residential, transportation, croplands, livestock, mining, oil and gas, forestry, and tourism/recreation), physical/climatic features (topography, soils, drainage, vegetation, temperature, precipitation, etc.) and natural disturbance (fire, insects, storms, etc.). It has been widely employed to explore future environmental trajectories and guide land use decisions in North and South America, Australia, India, and elsewhere^5,23^.

In this project, the model is used to simulate the key dynamics of terrestrial land uses and landscapes in the Kimberley region, and to generate output that is relevant to the interface between terrestrial and marine ecosystems. The model was calibrated with best available data after extensive consultation with Australian federal, state, and regional governments, research institutes, resource companies, and other stakeholders, and therefore incorporates standard assumptions for each sector (for details of data sources, see^24^). We then used the model to explore proposed future trajectories in human populations, settlements, mining, energy, croplands, livestock, tourism, and transportation, and generates spatial and temporal information on land use and natural disturbance regimes. The model applies user-specified rates of natural disturbance and change in population and human activity in successive time steps to generate changes in land use, vegetation, and other physical characteristics. Changes are assigned stochastically to plausible grid cells (e.g., increases in cropland where soils and hydrology are suitable; increases in urban footprint adjacent to existing urban areas; fire effects in appropriate vegetation types). We used the model to simulate 18 scenarios differing in intensity of human population growth, climate change, and precipitation regime, as described below in Section 4.2. Output for each scenario was inspected to ensure that overall rates of change matched the parameters for that scenario. The influence of these on marine and coastal systems can then be incorporated as forcing to the marine system model.

Ecopath with Ecosim (EwE; www.Ecopath.org) was used to characterise the trophic structure, ecosystem attributes and impact of fishing, other human uses and climate change on the marine environment. Ecopath is a mass-balance model that accounts for trophic interactions among organisms at multiple trophic levels by describing matter and energy flows^4^. Ecosim and Ecospace use the Ecopath model as initial conditions for temporal and spatial simulations of foodweb dynamics due to human activities and environmental drivers. The model covers an area of 316,966 km^2^ (17°34′S, 125°46′E) and includes nine marine parks located within 200 m depth contour (three Commonwealth and six state marine parks) as shown in Supplementary Fig. S1. Three of these marine parks (80 Miles Beach, Kimberley and Yawuru Nagulagun/Roebuck Bay) are located in Commonwealth waters (within 3–200 Nautical Miles from the coast) and provide protection for important marine habitats and species using two types of IUCN (International Union for Conservation of Nature) zoning: (1) National Park Zone (referred to as ‘MPAs’ in this study, which allows vessel transit, but no recreational/commercial fishing, anchoring or tourism) and (2) Multiple Use Zone (where recreational and commercial fishing is allowed, except trawl, net and longline gears; tourism and shipping are permitted). The other six marine parks in the model (80 Mile Beach, Lalang-garram/Camden Sound, Yawuru Nagulagun /Roebuck Bay, North Lalang-garram, Lalang-garram/Horizontal Falls, and North Kimberley) are located in Western Australia state waters (within 3 NM from the coast). Due to the size of the model domain, we only considered two zoning in the state marine parks: (1) Sanctuary Zones (areas where fishing, vessel transit and tourism are not permitted according to the IUCN-1 regulations, see details in^1^), and (2) Multiple Use Zones. Special Purpose Zone (IUCN VI) and Habitat Protection Zone (IUCN IV) were not included because they represented less than 0.1% of the model domain. In the model, the Multiple Use Zones, the National Park Zones and Sanctuary Zones cover ~90,000 km^2^ (~30% of the total marine area modelled) (Table 2).

EwE foodweb contains 59 functional groups (~110 species), including two non-living groups (terrestrial inputs and organic detritus). A number of single species functional groups were defined for species of commercial or recreational fishing significance (e.g. Barramundi (*L. calcarifer*), King threadfin (*Polydactylus macrochir*), Spanish mackerel (*Scomberomorus commerson*)). The model also represents marine mammals, sea birds, invertebrates and plants.

The EwE model was calibrated using time series (2010–2014) data of relative abundance estimates (catch per unit of effort CPUE) and catch data^25^ of the major target finfish species (Barramundi (*L. calcarifer*), King threadfin (*P. macrochir*), Goldband snapper (*Pristipomoides multidens*), Emperors (*Lethrinus* spp) and Spanish mackerel (*S. commerson*)) in the Kimberley. We adjusted estimates of predator-prey parameters that influence degree of density dependence and thus the rates of change in the predicted biomass of fished species^26^. In general, the predicted biomasses were within 20% of the observed values. We used Ecosim to evaluate the effects of climate change (including sea surface warming, sea level rise and changes in precipitation regimes), key land-based processes (modelled by ALCES), fishing and the overall impact of human presence in coastal areas (waste, pollution, infrastructure development and tourism).

The EwE models allowed us to explore the effects of different management options, such as controls on fishing effort and spatial closures related to MPAs. For this, we defined 15 indicators, which fall into five classes:

1. Meta groups: including target fish taxon (emperors, snappers and Threadfin); marine mammals, seabirds, and fish with trophic levels 2 to 3 (planktivorous fish, small reef associated, herbivorous fish).
2. Keystone species (defined as “relatively low biomass taxon or functional groups with a structuring role in the food web”^27^): sharks, snubfin dolphin, barramundi and large reef fishes.
3. Charismatic taxon which hold a particular economic, social or cultural value to stakeholder groups: corals (hard and soft corals), marine turtles and dugong.
4. Habitats: seagrass and mangroves.
5. System level indicators designed to reflect the state of the overall food web rather than of some of its components. Here we used the Total Divergence^7^, a modification of the ‘Kullback–Leibler’ distance^28^ which measures changes in relative biomass of taxa and functional groups while accounting for changes in overall biomass (see Supplementary Note 7, for further explanation).

### The scenarios

Based on stakeholder consultations that identified climate change and population growth/economic development as the key drivers of future change in the Kimberley region, we used these two factors in laying out the scenarios to be explored by our models. These two drivers define the Climate Change and Development axes of the Future Plane (Fig. 3b). We subdivide each axis into three levels of increasing pressure: low, medium and high. Because of uncertainty in how climate change may affect precipitation regimes in the region, and the significant impact that different precipitation regimes can have in terms of agricultural productivity and sediment runoff in the marine environment, each level of climate change pressure is further divided into two precipitation regimes, low and high. This results in 18 (6 by 3) scenarios, which allow for an exploration of the interplay between climate and development pressure into the future.

The climate change scenarios selected were based on the simulations produced by a near-global eddy-rich Ocean General Circulation Model – OFAM3, to downscale the future changes of global ocean circulation based on the Representative Concentration Pathways (RCP) 8.5 climate model projections^29^. The RCP8.5 is based on upon a revision and extension of the IPCC A2 scenario and represents a worst-case possible future climate trajectory under on-going high carbon emissions^15^. We adopted RCP8.5 (which projects average global warming of 2.0 °C (1.4 to 2.6 °C range) and mean sea level rise of 0.30 m (0.22 to 0.38 m range) by mid-century) as the High Climate Change scenario for our model simulations. Following communication with the authors of^29^ the Medium and Low Climate Change scenarios were obtained by scaling the output of the OFAM3 model by associating a radiative forcing of 4.5 W/m^2^ and 2.6 W/m^2^, respectively. These correspond to the RCP4.5 projections of 1.4 °C (0.9 to 2.0 °C range) warming and 0.26 m (0.19 to 0.33 m range) sea level rise and the RCP2.6 projections of 1.0 °C (0.4 to 1.6 °C range) warming and 0.24 m (0.17 to 0.32 m range) sea level rise, respectively^16^.

To model the impact of climate change on the marine food web, EwE was forced with the projected changes in biomass of exploited species in the Australia EEZ (relative to baseline: mean 1981–2000) under RCP 2.6 and RCP 8.5^30,31^. This forcing takes the form of time-series of annual multipliers of fish productivity, mortality and predator search rates in Ecosim. Lacking more detailed information, the mean of the forcing for scenarios RCP2.6 and RCP8.5 was used for scenario RCP4.5. In addition, published trajectories of simulated changes in pelagic and benthic primary producers^6,31^ were incorporated as time series of forcing functions affecting directly biological production. This allowed us to represent physical factors affecting the Kimberley food web. The forcing function modified the rate of consumption (Q/B) of consumers affecting growth rates and biomass production^4^.

To explore the potential effects of climate change on the terrestrial domain, the ALCES Kimberley simulator was forced with projected changes in temperature and precipitation as described under scenarios RCP 2.6, 4.5, and 8.5 provided by CLIM Systems. Temporal and spatial changes in temperature and precipitation were then used as forcing variables (through multiplicative modifiers) to assess their effects on sediment and nutrient transport rates relative to current and projected changes in land use sectors (crops, livestock, human population, tourism/recreation, mining, oil and gas). The specific approach used to describe these climate-induced modifiers is provided in^32^.

The Development scenarios accounted for many sectors, including population, tourism, infrastructure development, agriculture, aquaculture, transport, mining and Oil & Gas. These types of development are often correlated, and at the scale of this study, individual events often averaged out so that the broader trends resulting from their correlations were more important. Exact details about these scenarios, the rationale for their choice and the implications for each sector can be found in^32^.

We defined three development scenarios (Table 1). The scenarios were used to parameterize the ALCES model of land use. Development scenarios were incorporated into the EwE model indirectly, via the outputs of ALCES. We loaded the following time-series of ALCES outputs as reference data to force changes in Ecopath: (i) consumption rates (Q/B) and production rates (P/B); and in Ecosim changes involving: (i) vulnerability to predation, and (ii) natural mortality (M).

### The management strategies

Potential management strategies reflected a spectrum of political attitudes toward marine conservation. Our approach was based on the belief that while these strategies may be applied at the regional scale, they need to reflect both a regional and national scope, for several reasons. First, this project addressed a regional spatial scale and a multi-decade temporal scale. This prevented us from considering local, short-term interventions. Second, because of the national, iconic significance of the Kimberley environment, efforts to protect its marine environment cannot be disconnected from the overall national attitude towards conservation. Third, over the decades to 2050, this attitude may change considerably: it may oscillate towards or against more environmental conservation and may even reverse conservation values which we now consider unshakable.

In consultation with the WA Department of Biodiversity, Conservation and Attractions and the WA Department of Primary Industries and Regional Development, we defined five broad levels of regulation pressure which reflect political and social attitudes towards environmental conservation: ‘High’ (reflecting an increasing appetite for environmental conservation), ‘Medium’ (based around current regulations and expectations about proposed regulations currently in the pipeline), ‘Low’ (based around current regulations, in which the proposed regulations currently in the pipeline do not materialise), ‘Reversed’ (a turn in political and social mood which reverses most current conservation initiatives and reflects a society change in environmental priorities) and ‘Worst Case’ (the removal of most forms of regulation).

Within these broad levels of regulation pressure, we assume that interventions under management control are based around three broad management tools. The first tool consists of the existing and proposed marine parks, including the restrictions on the activities allowed in different zones within these parks. The second management tool consists of regulations on fishing (as one of the key pressures on marine living resources), which includes the total spawning biomass that is allowed to be taken, as well as bag and size limits for specific species. The third tool consists of regulating the impact of other human uses, such as tourism and mineral, oil and gas exploration and extraction. We assume that the political and social acceptance of different levels of regulations will impose a strong correlation in the use and implementation of the available management tools. Details of the five management strategies are presented in Table 2.

### Sensitivity analyses

Sensitivity analysis of EwE outputs is presented in Supplementary Note 8 and Supplementary Fig. S5.

## Supplementary information

Supplementary information.

## Data Availability

Metadata associated with this project can be viewed at http://marlin.csiro.au/geonetwork/srv/eng/search#!078ffe36-d5f4-0f56-e053-08114f8c04ed. Both Model input data and model simulation data are available at https://data.pawsey.org.au/public/?path=/WA%20Node%20Ocean%20Data%20Network/WAMSI2/KMRP/2.2/2.2.8/ALCES/Input_data.
