# Supplementary material for: Modelling regional futures at decadal scale: application to the Kimberley region
Source: Sci Rep. 2020 Jan 21;10:849. doi: 10.1038/s41598-019-56646-x (PMC6972922; doi:10.1038/s41598-019-56646-x)
Supplement: Supplementary file 1 — Supplementary information. [file 41598_2019_56646_MOESM1_ESM.pdf]

Modelling regional futures at decadal scale: application to the Kimberley region

Fabio Boschetti[1,*], Hector Lozano-Montes[1] & Brad Stelfox[2]

[1]Commonwealth Scientific and Industrial Organisation, Australia.

[2]ALCES Group, Calgary, Alberta, Canada

[*]Correspondence to Fabio.Boschetti@csiro.au

Supplementary information

1 Details of MPA network

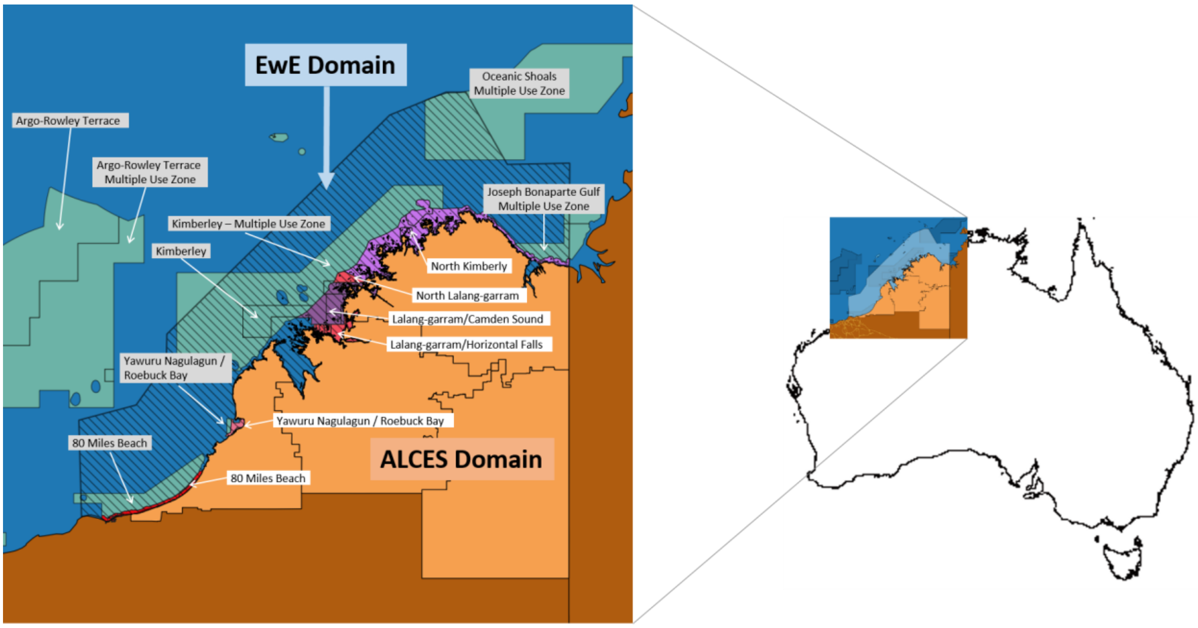

Supplementary Figure S 1. (left) Modelled area in the Kimberley region including i) Ecopath with Ecosim (EwE) marine domain (black diagonal lines) extending to ~200 m depth contour; and ii) ALCES land domain (in orange). The EwE model includes three marine parks (80 Miles Beach, Kimberley and Yawuru Nagulagun/Roebuck Bay) located in Commonwealth waters (3-200 NM; green polygons) which provide protection for important marine habitats and species in the region using two types of IUCN (International Union for Conservation of Nature) zoning: (1) National Park Zone (called in this study "MPA") (green polygons) which allows vessel transit, but no recreational/commercial fishing, anchoring or tourism and (2) Multiple Use Zone (where recreational and commercial fishing is allowed, except trawl, net and longline gears; tourism and transit are permitted) (green polygons). The model also includes other six marine parks (80 Mile Beach, Lalang-garram/Camden Sound, Yawuru Nagulagun /Roebuck Bay, North Lalang-garram, Lalang-garram/Horizontal Falls, and North Kimberley) located within 3 NM from the coast (in Western Australia state waters) (red/purple polygons). Due to the size of the model domain, we only considered two zoning in the state marine parks: (1) Sanctuary Zones (areas where fishing, shipping and tourism are not permitted according to the IUCN-1 regulations, see details in [1]) (not shown in the map because of the size of the model domain), and (2) Multiple Use Zones (red/purple areas). Special Purpose Zone (IUCN VI) and Habitat Protection Zone (IUCN IV) were not included in the spatial marine model because they represented less than 0.1% of the model domain. In the spatial marine model, the Multiple Use Zones, the National Park Zones and Sanctuary Zones cover ~90,000 km^2 (~30% of the total marine area modelled), including proportions of the Multiple Use Zones of

Oceanic Shoals Marine Park and Joseph Bonaparte Gulf Marine Park (part of the Northern Territories Network of Marine Parks). More details of zoning and managing activities can be found in [1].

2 EwE functional groups and their variation of input biomass data

Supplementary Table S 1. Ecopath with Ecosim (EwE) functional groups (first column) and variation of their input biomass based on changes of habitat, distribution, and productivity predicted by climate models, acidification processes, sea level rise and ALCES model. Whenever possible references are included in the table. In Ecosim (a temporal dynamic ecosystem model), we used different environmental and habitat parameters to simulate the predicted ALCES changes in estuaries, wetlands, and sediment yield transported by rivers to the ocean. We assumed that these changes in habitat quality and cover area could result in proportional effects on natural mortality, vulnerability to predation and relative feeding rates of species associated directly with the habitats affected. In the case of the climate change impacts, we used three levels of RCP emissions (see main document) to force the input biomass of target species based on the predicted trajectories of Australian fish stocks[2]

	Ecological group	Warming Impact (RCP 2.6) Low	Warming Impact (mean RCP 2.6 & RCP8.5) Medium	Warming Impact (RCP 8.5) High	Acidification Impact	Sea Level Rise	Climate Change (ALCES) Dryer	Climate Change (alces) Wetter
1	Humpback whales							
2	Baleen whales							
3	Dolphins	-7.4% [2]	-7.4% [2]	-7.4% [2]				
4	Dugong							
5	Coastal seabirds			+[3]			-13.4% (loss wetlands)	+17.9% (gain in wetlands & estuaries)
6	River birds						-13.4% (loss wetlands)	+17.9% (gain in wetlands & estuaries)
7	Migratory shorebirds			+[3]			-13.4% (loss wetlands & estuaries)	+17.9% (gain in wetlands & estuaries)
8	Lethrinids adults	-41.6% [2]	-44.2% [2]	-46.8% [2]	0[4] +[5] -[6]			
9	Lethrinids juv	-41.6% [2]	-44.2% [2]	-46.8% [2]	0[4] +[5] -[6]		-13.4% (loss wetlands & estuaries)	+17.9% (gain in wetlands & estuaries)
10	Snappers	-34.7% [2]	-38.4% [2]	-42.1% [2]	0[4] +[5] -[6]			
11	Adult Barramundi	-4.9% [2]	-5.8% [2]	-6.7% [2]	0[4] +[5] -[6]			
12	Juvenile Barramundi	-4.9% [2]	-5.8% [2]	-7.6% [2]	0[4] +[5] -[6]		-13.4% (loss wetlands & estuaries)	+17.9% (gain in wetlands/estuaries)
13	Threadfin	-4.8% [2]	-6.1% [2]	-7.4% [2]	0[4] +[5] -[6]			
14	Banana prawn	-29.6% [2]	-39% [2]	-48.4% [2]			-13.4% (loss wetlands & estuaries)	+17.9% (gain in wetlands/estuaries)

15	Tiger prawn	29.6% [2]	-39% [2]	-48.4% [2]			-9.5% (decreased sediment yield)	+10.5% (increased sediment yield)
16	Pearl oyster				-[4] -[5] -[6]		-9.5% (decreased sediment yield)	+10.5% (increased sediment yield)
17	Pelagic sharks	-23.8% [2]	-27.2% [2]	-30.6% [2]	0[4] +[5] -[6]			
18	Rays							
19	Billfishes and Tunas	-3.5% [2]	-5.8% [2]	-8.2% [2]	0[4] +[5] -[6]			
20	Demersal sharks	-22.5% [2]	-23.7% [2]	-24.9% [2]	0[4] +[5] -[6]			
21	Lizardfish				0[4] +[5] -[6]			
22	Large Reef Associated	-22.4% [2]	-22.8% [2]	-23.3% [2]	0[4] +[5] -[6]			
23	Small Reef Associated	-15.6% [2]	-18.3% [2]	-21.1% [2]	0[4] +[5] -[6]			
24	Reef Associated Pelagics	-25.2% [2]	-26.5% [2]	-27.9% [2]	0[4] +[5] -[6]			
25	Shallow demersal fish	-11.4% [2]	-14.8% [2]	-18.3% [2]	0[4] +[5] -[6]			
26	Planktivore fish	-4.6% [2]	-9% [2]	-13.4% [2]	0[4] +[5] -[6]			
27	Herbivorous fish	-17.9% [2]	-20.6% [2]	-23.3% [2]	0[4] +[5] -[6]			
28	Estuarine fish	-15.7% [2]	-18.5% [2]	-21.4% [2]			-13.4% (loss wetlands & estuaries)	+17.9% (gain in wetlands/estuaries)
29	Adult turtles					-[3]		
30	Green sea turtles			-[3]		-[3]		
31	Turtle hatchlings					-[3]		
32	Sea snakes							
33	Adult crocodile							
34	Juveniles crocodiles						-13.4% (loss wetlands)	+17.9% (gain in wetlands/estuaries)
35	Cane toad							
36	Reef building corals			-[3]	-[3] -[5] -[7] -[6] -[8]			
37	Non-reef building corals			-[3]	-[3] -[5] -[7] -[6] -[8]			
38	Squids							
39	Octopus							
40	Lobster	-4.2% [2]	-5.3% [2]	-6.4% [2]	-[6]			
41	Shells				-[4] -[5] -[7] -[9] -[6] -[8]			
42	Crabs	-26.5% [2]	-28.7% [2]	-31% [2]	-[6]		-13.4% (loss wetlands)	+17.9% (gain in

								wetlands/estuaries)
43	Epibenthos				-[4] -[5] -[7] -[9] -[6] -[8]		-9.5% (decreased sediment yield)	+10.5% (increased sediment yield)
44	Salps & Jellyfish			0 [10]				
45	Zooplankton			-[3]	-[3] -[4] -[7] -[9] -[8]			
46	Phytoplankton			-[3]	-[3] -[4] -[7] -[9] +[8]		-9.5% (decreased sediment yield)	+10.5% (increased sediment yield)
47	Macrophytes						-9.5% (decreased sediment yield)	+10.5% (increased sediment yield)
48	Seagrass				+[3] +[5] +[7] +[9] +[8]		-9.5% (decreased sediment yield)	+10.5% (increased sediment yield)
49	Mangroves				+ [3] +[7]	-	-13.4% (loss wetlands)	+17.9% (gain in wetlands/estuaries)
50	Detritus						-9.5% (decreased sediment yield)	+10.5% (increased sediment yield)

3 Responses of each indicator to each of the climate/development scenarios

Supplementary Figure S 2 shows the distribution of biomass changes per each modelled scenarios, where the bars map the change in biomass as *departure from the mean* of all scenarios. For each of the 3*3 scenarios, the pink or blue bars represent the dry (low precipitation) and wet (high precipitation) conditions, respectively. To simplify the analysis of the figure, only indicators whose final states change more than 40% compared to the median are included in this plot. The plot shows clearly the effect of the climate forcing in terms of warming, since the bars on the top row (High Climate Change) are mostly on the left hand side (<0) of the panels, while the bars on the bottom row (Low Climate Change) are all on the right hand side (>0) of the panels. The plot also shows the forcing due to the precipitation regimes, in particular in the middle row (Medium Climate Change) with the pink and blue bars pointing in opposite directions. On the contrary, the forcing due to socio-economic development is less clear.

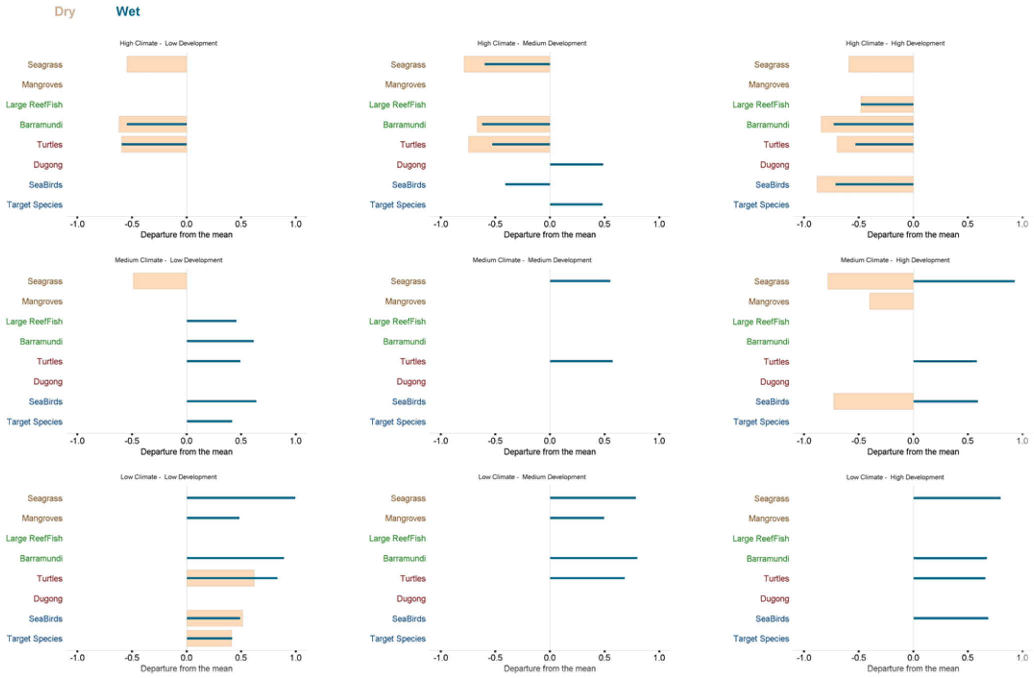

Supplementary Figure S 2. Bar plot of the state of all indicators (y axis) at the end of Ecosim simulation (2050), expressed as ratio of change over the value at the beginning of the simulation (2015). Only indicators whose final state change more than 40% compared to the mean of all scenarios are included in this plot.

4 Results of bootstrap analysis supporting importance of climate change over development

The cluster analysis of the scenario results suggests that climate change affects the model output more than the development scenarios. To assess the statistical significance of this results, we would need a much larger set of simulations than we can afford to run. As an alternative approach, we used bootstrapping ([11-13]) to generate a large number of surrogate EwE results and compared the output of the 18 scenarios so far discussed against this set of surrogate results. In particular:

1) We concatenated the biomasses of all living species (57) at the end of the simulation for each scenario, into a 18*57 matrix (one scenario per row, one species per column)
2) We generated a large (10^4) set of surrogate biomasses distributions. Each surrogate biomass distribution was generated by choosing, per each of the 57 columns (species), a random row and assigning the biomass value at that row to that species. This results in a 'realistic' set of biomasses values (since the value are the output of actual EwE runs), in which the relation between species biomass, as imposed by EwE dynamics, is lost.
3) For each of the (10^4) set of surrogate biomasses distributions, we compute the Total Divergence against a baseline EwE run. This results in a random distribution of 10^4 Total Divergence values.
4) Next, we compute the Total Divergence against a baseline EwE run for each of the 18 scenarios under analysis.
5) Finally, we add these 18 Total Divergence to the random distribution values and we sort the full set of 10018 Total Divergence in increasing order. The position of the Total Divergence of the 18 scenarios in this sorted list is a proxy for the statistical significance [11], that is, the closer to the extremes of the distribution these scenarios are, the more they statistically differ from the full set.

Four of the six high climate scenarios and two of the low climate scenarios are significantly different from the overall distribution, with a $p<0.05$, while five of the six high climate scenarios and four of

the low climate scenarios are significantly different from the overall distribution, with a p<0.1, where the high and low climate scenarios are at opposite extremes of the distribution.

5 Biomass changes for all indicators for each management strategy

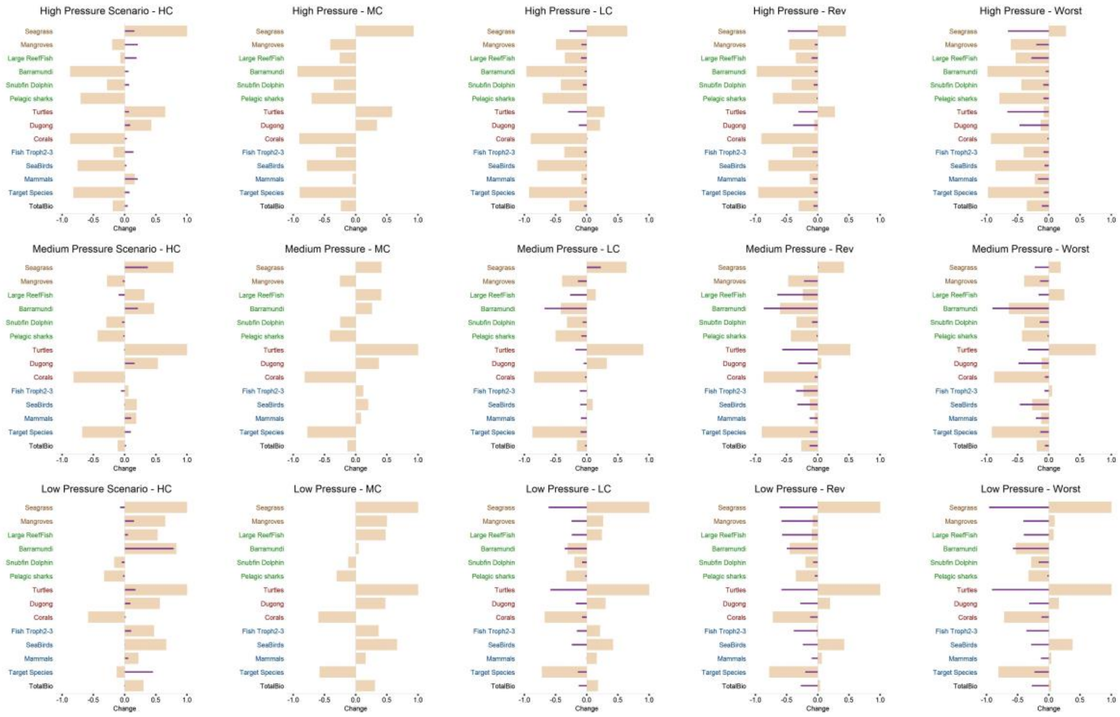

Supplementary Figure S 3. Bar plot of the state of all indicators (y axis) at the end of Ecospace simulations (2050). Beige bars show the ratio of change over the value at the beginning of the simulation (2015). Thin purple bars show contribution provided by a management strategy, compared to the contribution of the ‘Medium’ management strategy.

6 Relative impact of MPAs vs fishery regulation

We used the spatial dynamic model (Ecospace) to compare the model output biomass at the end of simulation in 2050 with and without the MPA network (see table below) under the same fishing regulations. The comparison was carried out for the high climate, high development, dry precipitation regime. Results are shown in Supplementary Figure S 4. The biomass for most species, including one of commercial interest, decreases in the absence of MPAs. The species which see the largest decline are barramundi, snappers, emperors and dugongs. The species which see an increase in biomass are their prey.

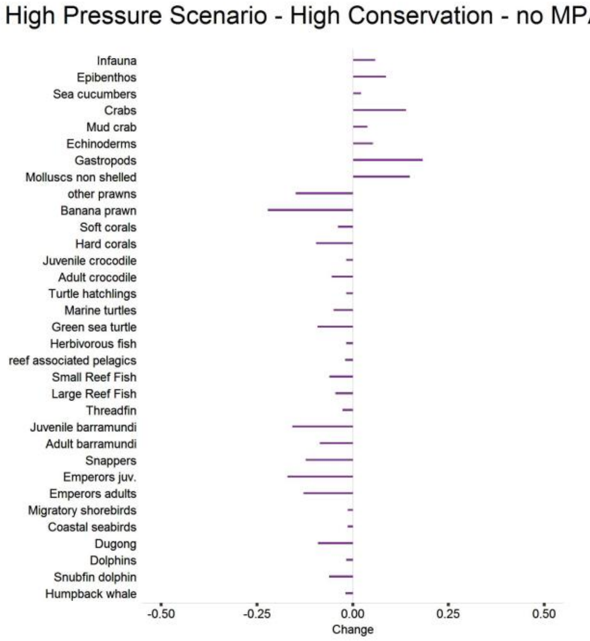

Supplementary Figure S 4. Ratio of change of the values at the end of the simulation of the high climate, high development, dry precipitation scenario with and without the MPA network.

7 Total Divergence

To cluster the 18 modelled scenarios for climate change and human development into natural groupings, we used the Total Divergence as a measure of scenario similarity. We need a measure which captures both changes in how the biomass is distributed between species as well as changes in total biomass.

Let's consider an ecological system made up of *n* functional groups. Each group has biomass B_i, $i=1..n$. At each time step *t*, we represent the system by its biomass distribution B_i^t.[1] Next, we normalise the biomass distribution $b_i = \frac{B_i}{\sum B_i}$. b_i can be understood as a probability distribution, that is, as the probability that a random unit of biomass in the ecosystem belongs to group *i*. Let's assume b_i is the output of an ecological model. At the beginning of the simulation ($t=t_0$) we have $b_i^{t_0}$. After running the model for Δt, the output biomass is $b_i^{t_1}$. We want to define a measure which reflects how much the system has changed during $\Delta t = t_1 - t_0$. A number of measures are available to compute difference between statistical distributions. An often used measure in information theory is the Kullback-Leibler divergence [15],

$$D_{KL}(b^{t_0}, b^{t_1}) = \sum_i b_i^{t_1} \log(\frac{b_i^{t_1}}{b_i^{t_0}}) \qquad (1)$$

D_{KL} is a weighed entropy and can be interpreted in several ways. One interpretation pertinent to the management of natural resources is that it represents the loss of information in studying the system via b^{t_0} when the system has changed to state b^{t_1}. Equivalently, it can be interpreted as the information gain in realising that the system has changed to b^{t_1}.

[1] At first sight, dismissing any information about the system structure (for example diet and competition relations between species) and limiting system description to time series of species abundance may appear as an over-simplification. The theoretical justification for this choice lays in Taken's theorem – 14 Takens, F. in *Dynamical systems and turbulence* Vol. 898 366-381 (Springer, 1981). – which shows that, under suitable conditions, the dynamics of the overall system can be recovered from the analysis of a single time series.

Nevertheless, the Kullback-Leibler divergence has a number of drawbacks which hamper its use in our application. First, it is not symmetric, $D_{KL}(b^{t_0}, b^{t_1}) \neq D_{KL}(b^{t_1}, b^{t_0})$ and as a result it does not define a proper distance metric. It also means that the divergence between 2 states depends on which one is seen first (although this can be make symmetric by replacing it with $\frac{D_{KL}(b^{t_0},b^{t_1})+ D_{KL}(b^{t_1},b^{t_0})}{2}$ [16]). A more serious drawback is that $D_{KL}(b^{t_0}, b^{t_1}) = \infty$ if any $b_i^{t_0} = 0$, which imply that there is no upper bound for $D_{KL}(b^{t_0}, b^{t_1})$. It also means that the symmetric version is not computable if either any $b_i^{t_0} = 0$ or any $b_i^{t_1} = 0$. To address these drawbacks, a number of alternative measures are reviewed in [16-18]. A particularly convenient option for our purpose is the Hellinger distance:

$$H(b^{t_0}, b^{t_1}) = \frac{1}{\sqrt{2}} \sqrt{\sum_{i=1}^{n} \left(\sqrt{b_i^{t_0}} - \sqrt{b_i^{t_1}} \right)^2} \qquad (2)$$

The Hellinger distance circumvents both drawbacks for the Kullback-Leibler divergence. Furthermore its maximum value is equal to one when $b_i^{t_0}$ has probability zero for all *i* for which $b_i^{t_1}$ has non-zero probability and vice versa. As we will see, this property is particular convent in our approach. As a result, in the rest of the discussion, we will use (2) in place of (1)[2].

Eq 2 measures how the biomass *distribution* at time t_1 changes relative to what it was at time t_0. As such, it can be understood as a measure of change in the ecosystem *biodiversity*. However, it says nothing of whether the total biomass $\sum B_i$ has changed. For example, if the biomass for each group decreases by 50%, $H(b^{T_0}, b^{T_1}) = 0$. To account for changes in total biomass we add a virtual group to our ecosystem. We call this group '*LostBiomass'* and we define it as $B_{n+1}^t = \sum_1^n B_i^P$-$\sum_1^n B_i^t$, where B_i^P represents the system *B* in an ideal unperturbed, pristine state. At each time *t*, *LostBiomass* includes the biomass not belonging to any group *i=1..n*. This can be interpreted as the biomass lost to the system. Because b_i, *i=1..n+1* must be a probability distribution, we must ensure that $B_{n+1} \geq 0$. We achieve this by assuming that the pristine system B_i^Phas higher total biomass that the perturbed states B^t. In practice, we do not need the full distribution B_i^P, but only the total biomass $B_{Tot}^P = \sum_1^n B_i^P$, which is constant, and then define $B_{n+1}^t = B_{Tot}^P - \sum_1^n B_i^t$.

8 Sensitivity analysis of the Ewe marine model

The Kimberley EwE model resulting from the mass-balancing process represents only one possible realization of this ecosystem. Model sensitivity to Ecopath input parameters (B, Q/B, P/B and diet) was carried out via Monte Carlo simulations in Ecosim (the time-dynamic model). The pedigree routine in Ecosim (see details in [20]) assesses the reliability of these input parameters based on data type and origin and determines the confidence of variation (CV) used in the Monte Carlo approach. This included 500 simulation runs of the high climate impact, high development, dry scenario, with parameters chosen from normal distributions centered on the initial input parameter estimates and CV determined by the model pedigree. This approach allowed us to identify the groups whose biomass changes the most as a function of input parameters and CV. In deceasing order, these are seagrass, phytoplankton, salps and jellyfish, zooplankton and macrophytes (Supplementary Figure S 5, left panel, also highlighted in yellow in the EwE foodweb). The Monte Carlo runs also provide indications about the overall trophic control in the system and suggest that the Kimberley marine food web is associated with bottom-up control because of larger sensitivity to changes in the biomass of the lower section of the food web (functional groups within trophic levels of 1 to 2.5).

---

[2] The Hellinger distance is not based on measures of entropy and as a result, technically, its use do not fall within the class of information-theoretical approaches. However, the Hellinger distance can be used to study information properties of mathematical spaces in special cases (see 19 Shemyakin, A. Hellinger Distance and Non-informative Priors. *Bayesian Anal.* **9**, 923-938, doi:10.1214/14-BA881 (2014).). To be accurate, we call it approach information-theory inspired.

In addition, the quality of the input data was also assessed in the mass-balancing of the EwE model. We computed the Ecotrophic Efficiency (EE), a measure of the proportion of production that is utilized in the system by predation or fishing (EE cannot exceed 1.0, that is, it is not possible to consume more than is produced). As a general rule, EE values near 1.0 are expected for groups whose production is consumed by predators or removed by the fishery while values near to 0.0 are expected for groups with low predation rates (e.g. top predators like sharks) or not targeted by a fishery. Checks on EE values, and thermodynamic and ecological rules of thumb [20] (i.e. slopes of biomass ratios, total production, mortality rates, and values of Ecotrophy Efficiency) are used to highlight groups in the pre-balanced model whose input parameters need to be adjusted within biologically plausible limits to achieve mass-balance of the flows in the food-web. We reduced predation mortality rates of groups with unrealistic EEs (juveniles of Lethirnids, pelagic fish, mud crabs, squids, deep demersal fishes, turtle hatchlings, Banana prawns, Large reef fishes among others) by identifying their main predators and adjusting diet matrices until EEs were <1.0. This diagnostic approach highlighted the groups with more reliable data and therefore whose model predictions have lower uncertainty. The two approaches adopted in this study, provided both an assessment of the effect of uncertainty in Ecopath input data as well as an identification of knowledge gaps of the foodweb elements.

Group name	% Change
Seagrass	30.16
Phytoplankton	29.36
Zooplankton	12.43
Salps and jellyfish	10.33
Macrophytes	6.66
Bivalves	5.69
Turtle hatchlings	5.42
Small Reef Fish	4.35
Herbivorous fish	3.60
Estuarine Fish	3.32
Echinoids	2.60
Mud crab	2.57
Infauna	2.38
Gastropods	2.15
Planktivore fish	1.83
Banana prawn	1.60

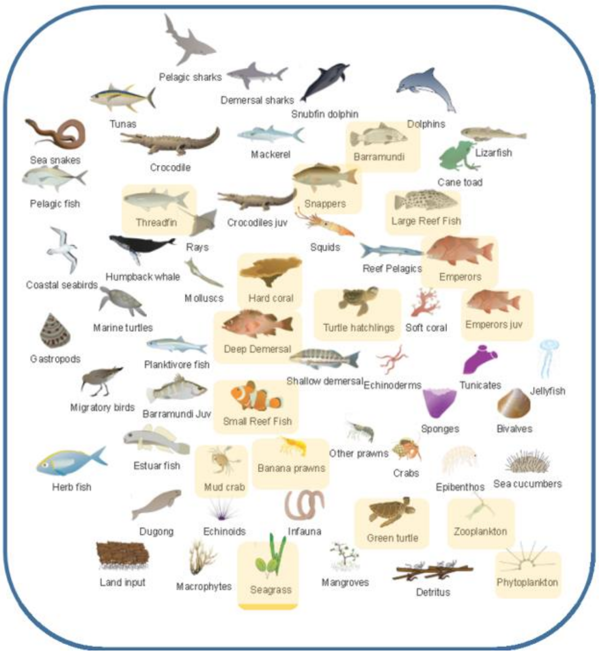

Supplementary Figure S 5. Sensitivity analysis of the input data in the EwE marine food-web (centre panel) was addressed by i) running Monte Carlo simulations in Ecosim with different coefficients of variation determined by the quality and origin of the input data and ii) by checking the Ecotrophic Efficiency values of the pre-balance model. The groups whose biomass changes the most as a function on input parameters and CV are presented in the left column and highlighted in the yellow in EwE foodwen in the middle panel. Values represent percentage change in biomass at the end of the simulation. The groups with the highest EE values are presented in the right column. These approaches highlight knowledge gaps and provide a better understanding of the overall trophic control in the system. Symbols courtesy of the Integration and Application Network (ian.umces.edu/symbols/)

9 Work Flowchart

Supplementary Figure S 6 shows the flowchart of the process we have used to carry out this research.

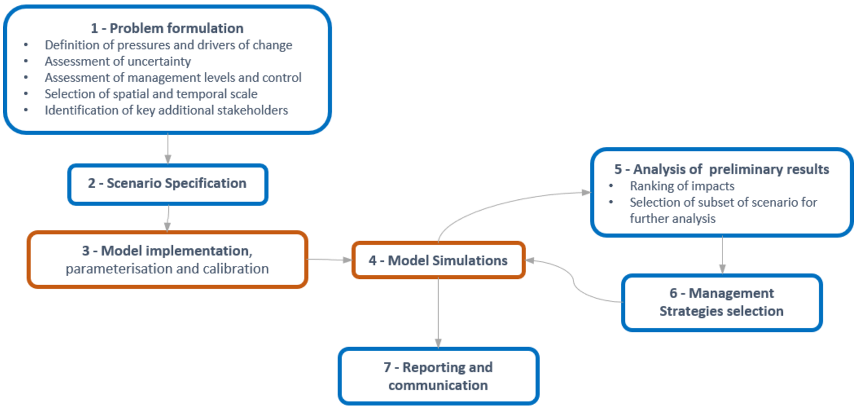

Supplementary Figure S 6. Flowchart of the process we have used to carry out this research. Blue boxes represent activities involving stakeholder input and interaction. Red boxes represent activities mostly involving computer modelling.

Box 1 represents the first step involving the problem formulation. This was carried out in collaboration with an initial set of stakeholders and led to agreement on the pressures and drivers of change expected to have the highest impact on the future of the Kimberley region. In turn, this led to an assessment of the level of uncertainty on the state of the system and on the processes acting on it and a discussion on the kind of levers and control available to the management authorities. This step also involved the identification of a larger set of stakeholders including institutes and researchers able to provide data for model parametrisation. The stakeholder team included both stakeholders familiar with modelling and the modelling team as well as stakeholders who had never interacted before this project.

Reaching a shared understanding on the most significant pressures, drivers of change and uncertainties was also necessary in order to select the appropriate temporal and spatial scale for the analysis as well as to realise that adaptive management could not be used as the only approach to this task, as discussed in the Introduction in the main document. The acknowledgment that the problem would benefit from being cast within a Future Studies approach led to the definition of the set of scenarios as discussed in Section 4.2 in the main document (Box 2). With a definition of the purpose, temporal and spatial scale of the analysis and the scenario to model, the team progressed to the model implementation, parameterisation and calibration (Box 3) (see Section 4.1 in the main document).

The initial set of model simulations allowed the team to identify climate as the main driver of change in the system and to select a subset of scenarios for further analysis (Box 4). At this stage, interaction with stakeholders helped identify a set of strategies available to manage the network of marine parks (Section 4.3 in the main document) (Box 6) for further model simulation. In the figure, blue boxes represent activities involving stakeholder input and interaction, while the red boxes represent activities mostly involving computer modelling. As can be seen, closed interaction with different stakeholder teams was carried out at all stages of the project.

Supplementary Figure S 6 also helps us obtain a rough picture of the relation between adaptive management and Future studies approaches as carried out in the project. While the project was carried out as a whole, and each activities benefitted by the team's appreciation of both approaches, it is easy to recognise the adaptive management/Management Strategy Evaluation (MSE)[21,22] cycle on the right (Boxes 4, 5 and 6) and the problem formulation following a Future studies approach on the left (Box 1 and 2).

10 Supplemental References

1 Parks, D. o. N. North-west Marine Parks Network Management Plan. Report No. ISBN: 978-0-9876152-3-7 (Director of National Parks, Canberra., 2018).

2 Fulton, E. A. *et al.* Decadal scale projection of changes in Australian fisheries stocks under climate change. (Fisheries Research and Development Corporation, Australia, 2018).

3 Poloczanska, E. S. *et al.* Climate change and Australian marine life. *Oceanography and marine biology* **45**, 407 (2007).

4 Fabry, V. J., Seibel, B. A., Feely, R. A. & Orr, J. C. Impacts of ocean acidification on marine fauna and ecosystem processes. *ICES Journal of Marine Science: Journal du Conseil* **65**, 414–432 (2008).

5 Kroeker, K. J. *et al.* Impacts of ocean acidification on marine organisms: quantifying sensitivities and interaction with warming. *Global Change Biol* **19**, 1884-1896, doi:10.1111/gcb.12179 (2013).

6 Wittmann, A. C. & Pörtner, H.-O. Sensitivities of extant animal taxa to ocean acidification. *Nature Climate Change* **3**, 995-1001, doi:10.1038/nclimate1982 (2013).

7 Guinotte, J. M. & Fabry, V. J. Ocean Acidification and Its Potential Effects on Marine Ecosystems. *Ann Ny Acad Sci* **1134**, 320-342, doi:10.1196/annals.1439.013 (2008).

8 Aze, T., Barry, J., Bellerby, R. & Diversity, S. o. t. C. o. B. *An updated synthesis of the impacts of ocean acidification on marine biodiversity*. (2014).

9 Doney, S. C., Fabry, V. J., Feely, R. A. & Kleypas, J. A. Ocean Acidification: The Other CO_2 Problem. *Annual Review of Marine Science* **1**, 169-192, doi:10.1146/annurev.marine.010908.163834 (2009).

10 Brotz, L., Cheung, W. W. L., Kleisner, K., Pakhomov, E. & Pauly, D. Increasing jellyfish populations: trends in Large Marine Ecosystems. *Hydrobiologia* **690**, 3-20, doi:10.1007/s10750-012-1039-7 (2012).

11 Unsworth, C. P., Cowper, M. R., McLaughlin, S. & Mulgrew, B. A new method to detect nonlinearity in a time-series: synthesizing surrogate data using a Kolmogorov–Smirnoff tested, hidden Markov model. *Physica D: Nonlinear Phenomena* **155**, 51–68 (2001).

12 Berry, K. J., Johnston, J. E., Mielke, P. W. & Johnston, L. A. Permutation methods. Part II. *Wiley Interdisciplinary Reviews: Computational Statistics* **10**, e1429, doi:10.1002/wics.1429 (2018).

13 Ojala, M. & Garriga, G. C. in *2009 Ninth IEEE International Conference on Data Mining (ICDM).* 908-913 (IEEE).

14 Takens, F. in *Dynamical systems and turbulence* Vol. 898 366-381 (Springer, 1981).

15 Hobson, A. *Kullback–Leibler divergence*. (Gordon and Breach, 1971).

16 Cha, S.-H. Comprehensive survey on distance/similarity measures between probability density functions. *City* **1**, 1 (2007).

17 Lin, J. Divergence measures based on the Shannon entropy. *Ieee T Inform Theory* **37**, 145–151 (1991).

18 Cichocki, A. & Amari, S.-i. Families of Alpha- Beta- and Gamma- Divergences: Flexible and Robust Measures of Similarities. *Entropy* **12**, 1532-1568, doi:10.3390/e12061532 (2010).

19 Shemyakin, A. Hellinger Distance and Non-informative Priors. *Bayesian Anal.* **9**, 923-938, doi:10.1214/14-BA881 (2014).

20 Christensen, V. & Walters, C. J. Ecopath with Ecosim: methods, capabilities and limitations. *Ecological modelling* **172**, 109–139 (2004).
21 Bunnefeld, N., Hoshino, E. & Milner-Gulland, E. J. Management strategy evaluation: a powerful tool for conservation? *Trends in Ecology and Evolution* **In Press** (2011).
22 Smith, A. D. M., Sainsbury, K. J. & Stevens, R. A. Implementing effective fisheries-management systems - management strategy evaluation and the Australian partnership approach. *ICES Journal of Marine Science* **56**, 967-979, doi:10.1006/jmsc.1999.0540 (1999).
